# Characterization and Biocontrol Potential of *Bacillus velezensis* FB-4 Against *Valsa* Canker of Korla Fragrant Pear

**DOI:** 10.3390/jof12050349

**Published:** 2026-05-08

**Authors:** Zhen Zhang, Zhe Wang, Qinyuan Xue, Jiahui Yu, Tailong Li, Lan Wang, Hongzu Feng

**Affiliations:** 1 College of Life Science and Technology, Tarim University, Alar 843300, China; 17591588959@163.com (Z.Z.); 949510@163.com (Z.W.); 1317926912@163.com (Q.X.); 15026192189@163.com (J.Y.); 18738725702@163.com (T.L.); 2 Key Laboratory of Integrated Pest Management (IPM) of Xinjiang Production and Construction Corps in Southern Xinjiang, Tarim University, Alar 843300, China; 3 The National and Local Joint Engineering Laboratory of High Efficiency and Superior-Quality Cultivation and Fruit Deep Processing Technology of Characteristic Fruit Trees in Southern Xinjiang, Tarim University, Alar 843300, China

**Keywords:** pear *Valsa* canker, *Bacillus velezensis*, biological control, endophyte, plant growth promotion

## Abstract

The occurrence of pear *Valsa* canker caused by *Cytospora pyri* poses a serious threat to the healthy and sustainable development of the Korla fragrant pear industry. To effectively control the disease, endophytic strains were isolated from the bark of Korla fragrant pear trees and screened for strong antagonistic activity against the pathogen. The selected strain was identified based on morphological characteristics and 16S rRNA phylogenetic analysis. Its biocontrol potential and functional traits were further evaluated, along with its growth-promoting ability assessed through in vitro tests and preliminary tomato pot experiments. Results showed that a total of 264 endophytic isolates were obtained from 200 pear tissue samples using dilution plating and tissue separation methods. Among them, strain FB-4 exhibited significant inhibition against *C. pyri*. Morphological observations and phylogenetic analysis identified the strain as *Bacillus velezensis*, named FB-4. The cell-free supernatant inhibited conidial germination and mycelial growth of the pathogen by 88.21% and 85.51%, respectively, and showed preventive and curative efficacies of 74.49% and 58.97% against pear *Valsa* canker. In vitro assays indicated that FB-4 could produce indole-3-acetic acid, protease, amylase, and cellulase, and demonstrated abilities to solubilize inorganic phosphate and synthesize siderophores. Inoculation with FB-4 bacterial suspension promoted the growth of tomato seedlings, with higher concentrations yielding more pronounced effects. In conclusion, strain FB-4 represents a dual-functional biocontrol agent with both antagonistic and plant-growth-promoting properties.

## 1. Introduction

Korla fragrant pear (*Pyrus sinkiangensis* Yü) is a representative premium cultivar within the Chinese pear germplasm and serves as a vital economic fruit tree and characteristic agricultural product in the Xinjiang region [1,2]. Renowned as a “rare treasure among pears,” its fruits are distinguished by their intense aroma and rich content of pharmacologically active compounds [3,4]. However, this cultivar exhibits high susceptibility to *Valsa* canker caused by *Cytospora* species (teleomorph *Valsa*), making the disease a major threat across all Korla fragrant pear production areas in Xinjiang [5,6,7]. Wang et al. identified *Cytospora pyri* as the predominant causal agent of Korla fragrant pear canker in the region [6]. The disease is now widespread and spreading rapidly, severely impacting both the yield and quality of the fragrant pears. Pear *Valsa* canker is distributed extensively across pear-growing regions in Asia, Europe, and North America [8,9]. In China’s major pear production areas, including the Northwest, Northeast, North China, and the old course of the Yellow River, the disease inflicts serious damage, with incidence rates in severely affected orchards reaching 77.8% to 100.0% [10]. Consequently, developing effective control measures to manage Korla fragrant pear *Valsa* canker has become a critical and urgent issue.

Over recent decades, the traditional philosophy of “emphasizing treatment over prevention” has proven inadequate for managing fruit tree *Valsa* canker under current conditions [11,12]. Contemporary control strategies should prioritize “prevention” over “therapy”, with core objectives focused on reducing pathogen inoculum in the field and enhancing tree vigor [13,14]. While complete eradication of the disease may not be feasible, implementing a series of “preventive” measures plays a positive role in disease management, contributing to yield increase and stabilization in Korla fragrant pear production [15]. At present, control of this disease relies primarily on chemical methods. However, most chemical treatments fail to exert effective action within the tree trunk, allowing the pathogen to develop unimpeded [16]. Furthermore, the potential toxicity and ecological risks associated with chemical residues are becoming increasingly concerning, driving the search for more sustainable alternative control strategies [17]. In this context, innovative approaches based on sustainable agricultural practices, such as biological control, have emerged as promising tools for managing Korla fragrant pear *Valsa* canker while mitigating the adverse impacts of conventional methods.

Biocontrol agents represent a promising strategy for managing *Valsa* canker caused by *C. pyri* in Korla fragrant pear. Compared to chemical pesticides, this approach offers advantages such as environmental friendliness, non-toxicity, high efficacy, and the ability to provide sustained protection within the host, making it one of the optimal choices for disease management. In recent years, substantial research on the biological control of Korla fragrant pear *Valsa* canker has been conducted by scholars both domestically and internationally. To date, several microorganisms have demonstrated potential for effective disease control [18,19,20]. For instance, Yuan et al., while investigating the biocontrol agent *Bacillus atrophaeus* HF1 against *Valsa pyri*, found that strain HF1 inhibited mycelial growth of the pathogen by 61.20% and induced abnormal hyphal development; Both its culture filtrate and volatile organic compounds antagonized the growth of *V. pyri* [20]. Song et al. were the first to report that dipicolinic acid (DPA) produced by *Bacillus subtilis* exhibited significant antifungal activity against various *Valsa* canker pathogens, including *V. pyri* [21]. Zhang et al. discovered that cell-free supernatant (CFS) from *Trichoderma virens* strongly inhibited the mycelial growth of *V. pyri*, causing treated hyphae to become deformed, constricted, and suffer oxidative damage [22]. Although these biocontrol agents show promising potential, only one is currently registered for use in China [20]. Therefore, further exploration of other stable and efficient antagonistic microbial control strategies holds significant value, as they could help alleviate the spread and outbreak of Korla fragrant pear *Valsa* canker.

The primary objective of this study was to isolate and identify novel antagonistic endophytes from the bark of Korla fragrant pear trees with high inhibitory activity against *Cytospora pyri*, the causal agent of *Valsa* canker. Specifically, we aimed to: (i) screen for endophytic isolates showing strong antagonistic effects; (ii) characterize the antimicrobial activity, biofunctional traits, and plant growth-promoting capacity of the most promising strain; and (iii) evaluate its potential as a biocontrol agent. This work seeks to expand the available microbial resources for the sustainable management of *Valsa* canker in Korla fragrant pear production.

## 2. Materials and Methods

### 2.1. Plant Samples and Culture Media

Plant samples: In July 2023, 25 healthy Korla fragrant pear trees were randomly selected from an orchard (40°54′52″ N, 81°12′23″ E) in Alaer City, the First Division of the Xinjiang Production and Construction Corps, China. From each tree, 8 pieces of bark tissue were collected, resulting in a total of 200 bark samples.

Culture media: The LB solid medium contained (per liter): 10 g tryptone, 5 g yeast extract, 5 g NaCl, and 18 g agar. LB liquid medium was prepared without agar. Potato Dextrose Agar (PDA) medium contained (per liter): 200 g potato, 20 g glucose, and 18 g agar. Potato Dextrose Broth (PDB) was prepared without agar. Media for detecting protease, nitrogen fixation, siderophore production, inorganic phosphate solubilization, organic phosphate solubilization, cellulase, amylase, and potassium solubilization were prepared according to the method described by Li et al. [23].

### 2.2. Isolation of Microorganisms from Korla Fragrant Pear Bark

Samples were first rinsed under running distilled water to remove surface debris. Surface sterilization was then performed by sequential immersion in 75% (*v*/*v*) ethanol for 1 min, 1% (*v*/*v*) sodium hypochlorite for 3 min, followed by three rinses with sterile distilled water. The final rinse water was plated on LB solid medium and incubated at 28 °C in the dark for 3 days to verify the effectiveness of the sterilization. The sterilized bark tissues were aseptically cut into 0.5 cm segments. Each segment was placed on PDA medium for isolating endophytic fungi. For endophytic bacteria isolation, 1 g of sterilized sample was ground into a homogenate with a sterile mortar and pestle, suspended in 10 mL of sterile water, and serially diluted (10^−1^, 10^−2^, 10^−3^). Aliquots (100 µL) of each dilution were spread onto LB solid medium. All plates were incubated at 28 °C in the dark for 3 days, with three replicates per dilution. Colonies with distinct morphologies were purified by repeated streaking. Pure cultures from the third streaking were preserved in 20% glycerol at −20 °C. Isolates were designated with a code: the first capital letter indicated the sampling location; the second letter indicated the taxonomic group (B for bacteria, F for fungi); and the following number represented the isolate sequence from that location.

### 2.3. Screening for Antagonistic Microorganisms

*Cytospora pyri*, the causal agent of Korla fragrant pear canker, was cultured on PDA plates at 26 °C in the dark for 3 days. A dual-culture plate assay was used for primary screening. A 5 mm mycelial plug of *C. pyri* was placed in the center of a fresh PDA plate. Four purified endophytic isolates were inoculated as 5 mm plugs at equidistant positions 2.5 cm from the pathogen plug. Plates inoculated with *C. pyri* alone served as the control. All plates were incubated at 26 °C in the dark for 5 days. When the mycelium of the control covered the plate, the colony diameter of *C. pyri* in both treated and control plates was measured using the cross-streak method. The inhibition rate was calculated as: Inhibition rate (%) = [(Control colony diameter − Treated colony diameter)/Control colony diameter] × 100%. Each treatment had three replicates. The isolate showing the highest inhibition rate was selected for subsequent experiments.

### 2.4. Identification of Antagonistic Strain

Morphological identification: The selected antagonistic strain was streaked on LB solid medium and incubated at 28 °C for 24 h. Colony morphology was observed. Morphological identification was performed according to Bergey’s Manual of Determinative Bacteriology and Handbook for Systematic Identification of Common Bacteria. Gram staining was performed following the method of Xu et al. [24] For scanning electron microscopy (SEM) observation, the strain was cultured on LB solid medium at 28 °C for 12 h. Sample preparation involved chemical fixation, dehydration, critical point drying, gold sputtering, and observation.

Molecular identification: Genomic DNA was extracted using a commercial bacterial DNA extraction kit (TIANGEN Biotech, Beijing, China) and a fungal DNA extraction kit (Solarbio, Beijing, China). The 16S rRNA gene was amplified by polymerase chain reaction (PCR) using universal bacterial primers 27F (5′-AGAGTTTGATCCTGGCTCAG-3′) and 1492R (5′-GGTTACCTTGTTACGACTT-3′). The fungi were amplified by PCR using the universal primers of fungi, ITS1 (5′-TCCGTAGGTGAACCTGCGG-3′) and ITS4 (5′-TCCTCCGCTTATTGATATGC-3′). The purified PCR product was sequenced by Sangon Biotech (Shanghai) Co., Ltd., Shanghai, China. The obtained gene sequence was assembled and submitted to the GenBank database to obtain an accession number. For phylogenetic analysis, the sequence of antagonistic strain FB-4 was compared with reference type strain sequences using the BLASTn algorithm on the NCBI website (https://blast.ncbi.nlm.nih.gov, accessed on 10 February 2025). Multiple sequence alignment was performed, and a phylogenetic tree was constructed using the Neighbor-Joining method in MEGA software (version 7.0). Evolutionary distances were computed using the Kimura 2-parameter model, and the robustness of the tree topology was assessed by bootstrap analysis with 1000 replicates.

### 2.5. Determination of Inhibitory Effects of Cell-Free Supernatant and Volatile Compounds on Mycelial Growth

The Oxford cup method was used for quantitative assessment. Bacterial culture was prepared by inoculating the strain into LB liquid medium and incubating at 28 °C with shaking at 120 rpm for 48 h. The culture broth was centrifuged at 12,000× *g* for 5 min. The supernatant was filter-sterilized (0.22 µm pore size) to obtain the cell-free supernatant (CFS). The pellet was resuspended in sterile saline to prepare a bacterial suspension (1 × 10^8^ CFU/mL). For the assay, a *C. pyri* mycelial plug was placed in the center of a PDA plate. A sterile Oxford cup was placed 2.5 cm from the plate edge, and 200 µL of CFS was added to the cup. An equal volume of sterile LB broth served as the negative control. All plates were incubated at 26 °C for 5 days before measuring the colony diameter. The inhibition rate was calculated as described in Section 2.3. Each treatment had three replicates.

For the volatile compound assay, 100 µL of the bacterial suspension (1 × 10^8^ CFU/mL) was spread evenly on an LB agar plate and incubated at 28 °C for 2 days. A *C. pyri* plug was then placed in the center of a PDA plate. This PDA plate was inverted and sealed face-to-face with the LB plate using Parafilm, creating a dual-compartment system. The assembly was incubated for an additional 5 days. An LB plate without the antagonist served as the control. The pathogen colony diameter was measured, and the inhibition rate was calculated.

### 2.6. Effect of Cell-Free Supernatant on Conidial Germination

One-year-old Korla fragrant pear twigs in good condition were cut into 15 cm segments, washed, surface-sterilized (1 min in 75% ethanol, 3 min in 1% NaOCl), rinsed with sterile water, air-dried, and sealed at both ends with paraffin. A 5 mm deep hole was made in the xylem at the center of each segment. A 5 mm mycelial plug from a 3-day-old *C. pyri* culture was inoculated into each hole. The twigs were incubated at 26 °C in the dark under humid conditions. After conidial cirri developed on the lesions, they were scraped off with a sterile blade into 50 mL of sterile PDB, mixed thoroughly, and filtered through two layers of sterile gauze. The conidial concentration was adjusted to 1 × 10^8^ conidia/mL using a hemocytometer (Shanghai Qiujing Biochemical Reagent and Instrument Co., Ltd., Shanghai, China), resulting in the conidial suspension.

The CFS (from Section 2.5) was diluted 10-, 100-, and 1000-fold with LB broth. A 100 µL aliquot of each dilution was added to a sterile 1.5 mL centrifuge tube, followed by 100 µL of the conidial suspension (1 × 10^8^ conidia/mL). A control tube contained 100 µL conidial suspension and 100 µL sterile LB broth. Tubes were incubated at 26 °C for 36 h. Germination was observed under an optical microscope. The number of germinated conidia in five random fields (20 conidia per field) was counted for each replicate. The conidial germination inhibition rate was calculated as: Inhibition rate (%) = [(Number germinated in control − Number germinated in treatment)/Number germinated in control] × 100%. Each treatment had three replicates.

### 2.7. Inhibitory Effect of Cell-Free Supernatant at Various Concentrations

The inhibitory effect of *Bacillus velezensis* FB-4 CFS (prepared as described in Section 2.5) was evaluated by measuring mycelial biomass. A 5 mm mycelial plug of *C. pyri* was inoculated into potato dextrose broth (PDB) amended with 0, 2%, 4%, 6%, 8%, or 10% (*v*/*v*) FB-4 CFS. Cultures were incubated at 26 °C with shaking at 120 rpm for 5 days. The mycelia were then harvested, dried, and weighed. Each treatment included five replicates.

### 2.8. Efficacy of Cell-Free Supernatant Against Canker on Detached Twigs

One-year-old Korla fragrant pear twigs were prepared as in Section 2.6. A 5 mm deep hole was made in the xylem at the center of each segment. Four treatments were applied: (1) Protective effect: 50 µL of CFS was added to the hole, incubated at 26 °C for 1 day, then a *C. pyri* plug was inoculated. (2) Curative effect: A *C. pyri* plug was inoculated first, incubated for 1 day, removed, and then 50 µL of CFS was added. (3) Pathogen control: Inoculated with a *C. pyri* plug only. (4) Blank control: Inoculated with a sterile PDA plug only. Each treatment had five replicates. All twigs were incubated at 26 °C in the dark under humid conditions for 7 days. Lesion length was measured, and the control efficacy was calculated as: Efficacy (%) = [(Lesion length in pathogen control − Lesion length in treatment)/Lesion length in pathogen control] × 100%.

### 2.9. Analysis of Volatile Organic Compounds (VOCs) Produced by Bacillus velezensis FB-4 Using SPME–GC–MS

Headspace solid-phase microextraction (HS-SPME): Molten LB agar medium was dispensed into headspace vials and allowed to solidify. After cooling, 100 μL of a cell suspension of *B. velezensis* FB-4 (1 × 10^8^ CFU mL^−1^) was spread evenly onto the agar surface. Vials without bacterial inoculation served as controls. All vials were incubated at 28 °C. Volatile compounds were extracted using a 50/30 μm DVB/CAR/PDMS SPME fiber at 40 °C for 40 min.

SPME–GC–MS conditions: Chromatographic separation was performed on a DB-5MS capillary column. The injector temperature was set to 250 °C, and the analysis was carried out in splitless mode. The column temperature program was as follows: initial temperature of 40 °C held for 1 min, then increased at 6 °C·min^−1^ to 160 °C and held for 4 min, followed by an increase at 10 °C·min^−1^ to 210 °C and held for 5 min, and finally ramped to 220 °C and held for 2 min. Nitrogen was used as the carrier gas at a flow rate of 1.2 mL·min^−1^. The fiber was desorbed in the injector for 5 min.

Mass spectrometry conditions: Electron ionization (EI) was performed at 70 eV. The ion source temperature was 230 °C, the quadrupole temperature was 150 °C, and the transfer line temperature was 280 °C. Data were acquired in full-scan mode over a mass-to-charge ratio (*m*/*z*) range of 30–400. Data acquisition and processing were carried out using Agilent MassHunter software (version 10.0). Volatile compounds were identified by comparing their mass spectra with those in the NIST reference library, and assignments were confirmed based on the similarity scores generated by the software.

### 2.10. Determination of Biofunctional Characteristics

Indole-3-acetic acid (IAA) production: IAA production was quantified using the Salkowski colorimetric method. The strain was inoculated into LB liquid medium supplemented with L-tryptophan (200 mg/L) and incubated at 26 °C with shaking (120 rpm) for 96 h. The culture broth was centrifuged at 12,000× *g* for 10 min. One milliliter of supernatant was mixed with an equal volume of Salkowski reagent (50 mL of 35% HClO_4_ containing 1 mL of 0.5 M FeCl_3_) and incubated in the dark at room temperature for 30 min. A pink color indicated IAA production. Absorbance was measured at 530 nm. The IAA concentration was determined using a standard curve prepared with pure IAA (0, 0.5, 1, 5, 10, 15, 20, 25 mg/L). The experiment was performed with three biological replicates.

Other plant growth-promoting traits: Qualitative assays were performed on specific solid detection media. The strain was spot-inoculated onto media for inorganic phosphate solubilization and bacterial organic phosphate solubilization, and incubated at 28 °C for 7 days; a clear halo indicated positive activity. The ability to produce amylase, protease, siderophores, and cellulase, as well as nitrogen fixation and potassium solubilization, was assessed using the methods described in reference. The strain was spot-inoculated onto the respective solid detection media. Plates were incubated at 28 °C for 7 days, and the formation of a clear or colored halo around the colony indicated positive activity.

### 2.11. Assessment of Plant Growth-Promoting Capacity

Seed germination assay: Tomato seeds were surface-sterilized and soaked in the bacterial suspension (from Section 2.5) for 4 h. Seeds soaked in sterile water served as the control. Seeds were then placed on sterile moist filter paper in Petri dishes and incubated at 26 °C in the dark. Each treatment had five replicates, with 80 seeds per replicate. The number of germinated seeds was recorded after 72 h. The germination rate was calculated as: Germination rate (%) = (Number of germinated seeds/Total number of seeds) × 100.

Pot experiment with tomato seedlings: Tomato seedlings at the 3–5 true leaf stage with uniform growth were selected. For the treatment groups, 5 mL of bacterial suspension at three concentration levels—1 × (1 × 10^8^ CFU/mL), 10 × (1 × 10^9^ CFU/mL), and 50 × (5 × 10^9^ CFU/mL)—was applied to the rhizosphere soil of each seedling. Control seedlings received an equal volume of sterile water. Plants were grown under controlled conditions for 20 days, then carefully uprooted. Plant growth was evaluated by measuring plant height, fresh weight, and dry weight. Each treatment had 12 replicates.

### 2.12. Data Analysis

All experimental data were analyzed using SPSS Statistics software (Version 27.0, IBM Corp., Armonk, NY, USA). For experiments involving multiple treatments, one-way analysis of variance (ANOVA) was performed. When ANOVA indicated significant effects, post hoc comparisons were conducted using Tukey’s Honestly Significant Difference (HSD) test at a 95% confidence level (*p* < 0.05). Data are presented as the mean ± standard deviation (SD) of biological replicates.

## 3. Results

### 3.1. Isolation and Screening of Antagonistic Endophytes

A total of 264 endophytic isolates were obtained from 200 healthy pear tissue samples using tissue isolation and dilution plating methods. Preliminary screening via a dual-culture assay identified 19 bacterial and 2 fungal isolates that exhibited antagonistic activity against *Cytospora pyri*, the causal agent of Korla fragrant pear *Valsa* canker, with inhibition rates ranging from 63.31% to 85.51% (Table 1). BLAST (https://blast.ncbi.nlm.nih.gov, accessed on 21 January 2025) analysis revealed that all antagonistic isolates belonged to four phyla and eight genera. At the phylum level, the isolates were distributed among Firmicutes, Actinomycetota, Pseudomonadota, and Ascomycota. Specifically, Firmicutes contained *Bacillus* (seven species), *Paenibacillus* (one species), and *Metabacillus* (one species); Actinomycetota contained *Streptomyces* (four species); Pseudomonadota contained *Pseudomonas* (two species) and *Stenotrophomonas* (one species); and Ascomycota contained *Aspergillus* (one species) and *Chaetomium* (one species). Among the 21 antagonistic isolates, Firmicutes were predominant (12 isolates, 57.1%). At the genus level, *Bacillus* accounted for the highest proportion (10 isolates, 47.6%), followed by *Streptomyces* (4 isolates, 19.0%). The nucleotide sequences of all strains have been deposited in the NCBI GenBank database, and their similarity scores with reference sequences were at or close to 100%. Among these, the bacterial strain FB-4 showed the strongest antagonistic effect, suppressing mycelial growth by 85.51%. Consequently, strain FB-4 was selected for further investigation.

### 3.2. Identification and Characterization of Antagonistic Strain FB-4

**Figure 1:**
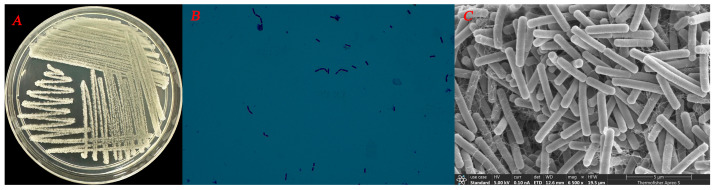
Morphology of strain FB-4 in culture. (**A**) Colony morphology of FB-4 on LB agar at 12 h, (**B**) Morphology on Gram stain, (**C**) Morphology under scanning electron microscope.

**Figure 2:**
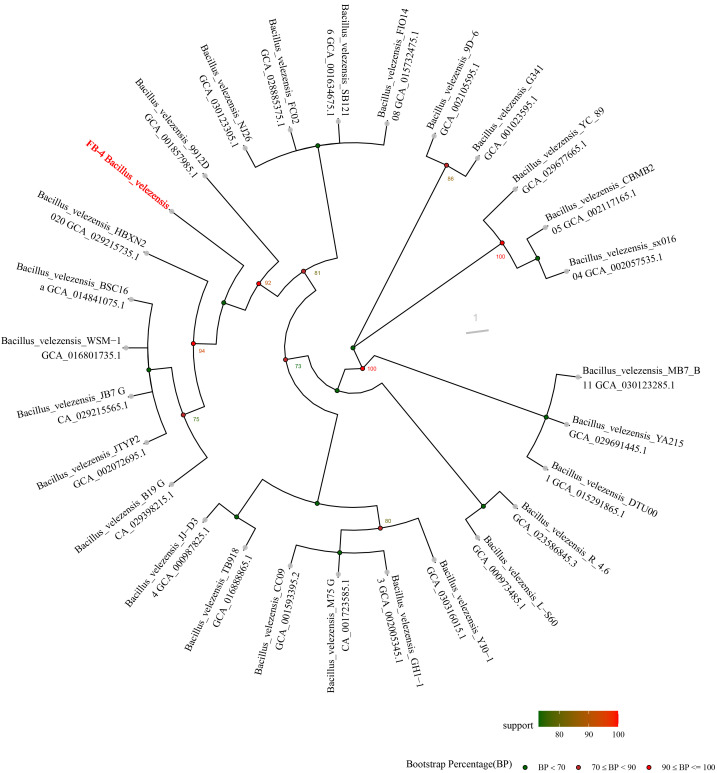
Phylogenetic tree based on 16 S rRNA gene sequences. Note: Colored dots indicate bootstrap support value ranges (detailed in legend inside the figure). The strain sequenced in this study (*Bacillus velezensis* FB‑4) is highlighted in red text.

Strain FB-4 formed pale yellow, slimy, translucent, convex colonies with smooth margins on LB agar after 24 h at 28 °C (Figure 1A). Light microscopy revealed that the cells were Gram-positive rods (Figure 1B). Scanning electron microscopy further confirmed the rod-shaped morphology, with cells measuring approximately 3.87 µm in length and 0.59 µm in width, and indicated the presence of an extracellular matrix (Figure 1C). Physiological and biochemical characterization showed that strain FB-4 was positive for Voges-Proskauer (VP) reaction, nitrate reduction, citrate utilization, and gelatin liquefaction, but negative for urease activity (Table 2). The strain utilized sucrose, xylose, maltose, and mannitol as carbon sources, but failed to grow in the presence of 7% (*w*/*v*) NaCl. Phylogenetic analysis based on the partial 16S rRNA sequence (GenBank accession no.: PX690612) placed strain FB-4 within the *Bacillus velezensis* clade (Figure 2). Based on these morphological, physiological, biochemical, and phylogenetic characteristics, strain FB-4 was identified as *Bacillus velezensis*.

### 3.3. Antifungal Activity of Strain FB-4 Against Cytospora pyri

**Figure 3:**
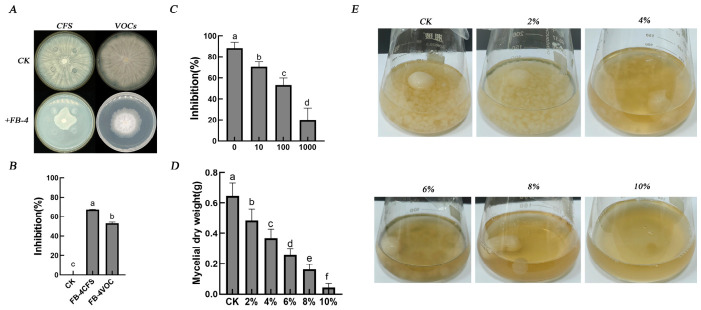
Inhibitory effects of *Bacillus velezensis* FB-4 on *Cytospora pyri*. (**A**) Colony morphology of *C. pyri* on PDA plates after 5 days of exposure to cell-free supernatant or volatile organic compounds (VOCs) from strain FB-4, compared with the untreated control. (**B**) Inhibition rates of mycelial growth following treatment with CFS or VOCs from strain FB-4. (**C**) Inhibition rates of spore germination by CFS of *B. velezensis* FB-4. (**D**) Mycelial dry weight of *C. pyri* after treatment with the indicated concentrations of CFS. Control (CK) cultures contained no CFS. (**E**) Morphology and size of mycelial pellets in PDB liquid culture containing increasing concentrations of CFS. Data are presented as mean ± SD (n = 3). Different lowercase letters indicate statistically significant differences (*p* < 0.05) according to Tukey’s HSD test.

The antimicrobial activity of the CFS from *Bacillus velezensis* FB-4 was determined using the Oxford cup assay. Compared to the blank control, the CFS significantly inhibited the mycelial growth of *C. pyri*, resulting in an inhibition rate of 67.12%. The inhibitory effect of volatile metabolites produced by strain FB-4 was assessed via a dual-plate sealed assay, which showed an inhibition rate of 53.19% against the pathogen (Figure 3A,B).

The CFS of *B. velezensis* FB-4, tested at different dilution levels, effectively inhibited the conidial germination of *C. pyri* in a dilution-dependent manner. The strongest suppression was observed with undiluted CFS (0× dilution), showing an inhibition rate of 88.21%. In contrast, the inhibitory activity decreased to 19.95% when the CFS was diluted 1000-fold (Figure 3C).

In liquid PDB medium, the presence of *B. velezensis* FB-4 CFS markedly suppressed the vegetative growth of the pathogen. The mycelial pellets in treated cultures were visibly larger and looser compared to the control (Figure 3E). This suppression was concentration-dependent, culminating in a 93.2% reduction in mycelial dry weight at the 10% CFS concentration relative to the control (Figure 3D).

### 3.4. Control Efficacy of Bacillus velezensis FB-4 Against Korla Fragrant Pear Valsa Canker

**Figure 4:**
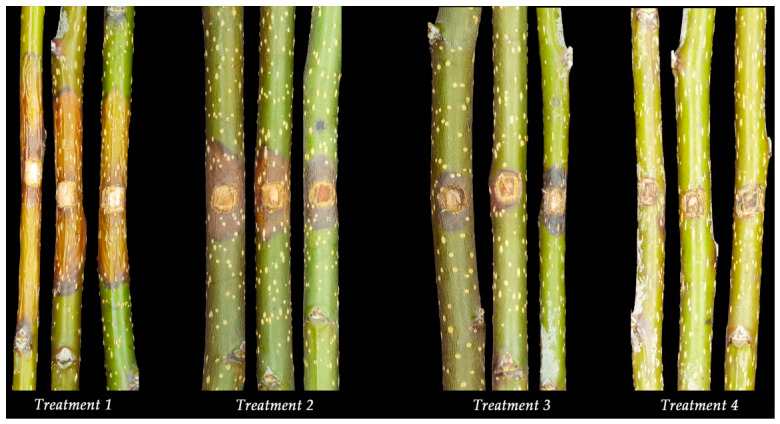
Lesion development on detached branches of Korla fragrant pear under different treatments. (Treatment 1) Inoculation with *Cytospora pyri* only; (Treatment 2) Inoculation with *C. pyri* followed by application of CFS from *Bacillus velezensis* FB-4; (Treatment 3) Application of CFS from antagonistic *B. velezensis* FB-4 followed by inoculation with *C. pyri*; (Treatment 4) Mock-inoculated control (PDA medium only).

The detached twig assay showed that no lesions developed in the blank control inoculated only with PDA medium. In contrast, twigs inoculated solely with *Cytospora pyri* exhibited significant lesion development, with an average length of 62.71 mm. In the protective treatment (application of *B. velezensis* FB-4 CFS followed by pathogen inoculation), lesion length was significantly reduced to 15.78 mm, corresponding to a control efficacy of 74.49%. In the curative treatment (pathogen inoculation followed by application of FB-4 CFS), the lesion length reached 25.57 mm, with an efficacy of 58.97% (Figure 4 and Table 3). These results demonstrate that the CFS of *B. velezensis* FB-4 possesses significant control efficacy against Korla fragrant pear *Valsa* canker and can effectively suppress lesion expansion.

### 3.5. Identification of Bacillus velezensis FB-4 VOCs

The VOCs produced by *Bacillus velezensis* FB-4 were analyzed using SPME–GC–MS. By comparing the mass spectra with the NIST library (Supplementary Materials S1 and S2) and subtracting background signals from the control (LB medium without bacterial inoculation), three compounds with potential antifungal activity against phytopathogenic fungi were identified: acetaldehyde, hexadecane, and 3-hydroxydodecanoic acid (Table 4). These VOCs are likely the primary bioactive components contributing to the antifungal activity of strain FB-4.

### 3.6. Plant Growth-Promoting Traits and Enzymatic Activities of Bacillus velezensis FB-4

**Figure 5:**
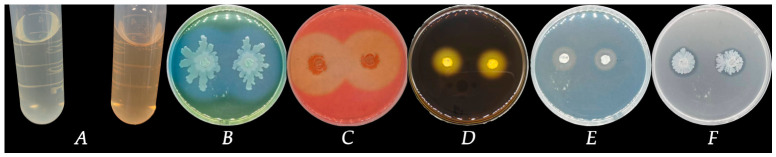
Plant growth-promoting and biocontrol traits of *Bacillus velezensis* FB-4. (**A**) Colorimetric detection of indole-3-acetic acid (IAA) production using Salkowski reagent. A pink color developed in the FB-4 culture supernatant (**right**) compared to the uninoculated LB medium control (**left**). (**B**–**F**) Qualitative plate assays showing the production of (**B**) protease (casein agar), (**C**) cellulase (CMC agar), (**D**) amylase (starch agar), (**E**) siderophores (CAS agar), and (**F**) phosphate solubilization (NBRIP agar). Positive activity is indicated by a halo surrounding the bacterial colony. Control plates inoculated with sterile LB medium showed no halos.

*Bacillus velezensis* FB-4 was evaluated for its plant growth-promoting potential. Quantitative analysis revealed that the strain produced IAA at a concentration of 120.83 mg/L (Figure 5A), as determined using a standard curve. Furthermore, the strain demonstrated multiple enzymatic and solubilization activities on specific solid media. Clear hydrolysis zones were observed around the bacterial growth on casein agar (protease activity), CMC agar (cellulase activity), starch agar (amylase activity), CAS agar (siderophore production), and Organo-phosphorus Medium (phosphate solubilization) (Figure 5B–F).

### 3.7. In Vivo Plant Growth-Promoting Effects of Bacillus velezensis FB-4

**Figure 6:**
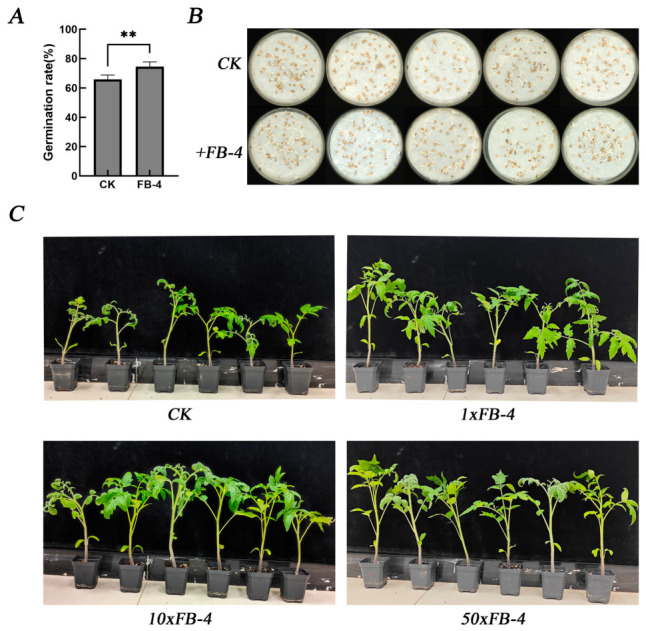
Plant growth promotion by strain FB-4 in tomato. (**A**) Germination rate of tomato seeds treated with FB-4 bacterial suspension (1 × 10^8^ CFU/mL) or sterile water (control). (**B**) Representative image of seed germination after 72 h. (**C**) Growth performance of tomato seedlings after soil drenching with FB-4 cell suspension at three concentration levels: 1× (1 × 10^8^ CFU/mL), 10× (1 × 10^9^ CFU/mL), and 50× (5 × 10^9^ CFU/mL), or sterile water (control), for 20 days. “**” denotes a statistically significant difference at *p* < 0.01.

The plant growth-promoting potential of *Bacillus velezensis* FB-4 was validated through seed germination and pot experiments of tomato. Treatment with the FB-4 bacterial suspension significantly enhanced the germination rate of tomato seeds, achieving 74.5% compared to 65.75% in the control (CK), representing an 8.75% increase (Figure 6A,B). In pot experiments, the application of FB-4 cell suspensions to tomato seedlings significantly improved growth metrics in a concentration-dependent manner (Table 5, Figure 6C). Plant height increased from 13.95 cm in the control to 16.89 cm (1×), 19.02 cm (10×), and 20.83 cm (50×) in treated plants. A concomitant increase in total plant biomass was also observed across all treatment groups.

## 4. Discussion

With the advancement of agricultural industrialization in China, regionally distinctive agricultural products are increasingly developing toward large-scale production. In this context, Korla fragrant pear has established concentrated and contiguous high-quality production bases [25]. Pear *Valsa* canker caused by *Cytospora* species was first reported in Japan in 1903 and subsequently emerged in regions such as New Mexico, USA (1925) and Kazakhstan (1972), remaining a global threat to pear production [26,27,28]. Bacteria of the genus *Bacillus* are often regarded as ideal candidates for commercial biocontrol agents due to their biocontrol potential [8]. In recent years, with the development of genomic sequencing technologies, *Bacillus velezensis* has attracted considerable attention for its remarkable antagonistic activity against plant pathogens [29,30,31]. This bacterium possesses unique biosynthetic gene clusters for secondary metabolites, providing a genetic basis for its outstanding biocontrol functions. Its genome contains various nonribosomal peptide synthetase and polyketide synthase gene clusters, enabling the synthesis of structurally diverse antimicrobial compounds [29,32]. Its antagonistic mechanisms primarily include the secretion of antimicrobial metabolites, competition for niches and nutrients, induction of plant systemic resistance, and promotion of plant growth [33].

In this study, 264 endophytic isolates were obtained from healthy Korla fragrant pear twigs using dilution plating and tissue isolation methods. Screening via a dual-culture assay identified 19 bacterial and 2 fungal isolates that exhibited significant antagonistic activity against the pear *Valsa* canker pathogen *Cytospora pyri*. Based on morphological observation, physiological and biochemical characterization, and 16S rRNA phylogenetic analysis, the most antagonistic strain, FB-4, was identified as *Bacillus velezensis*. Using high-throughput sequencing, Tang et al. [34] also reported high microbial diversity in the bark of healthy Korla fragrant pear trees and revealed the presence of numerous microbial taxa with potential biocontrol activity. *B. velezensis* has demonstrated significant antifungal capacity against various plant pathogenic fungi. For instance, strain B5 has shown efficacy in suppressing cabbage wilt caused by *Fusarium oxysporum* [35].

Further antagonistic assays were conducted using the cell-free supernatant and volatile organic compounds of *B. velezensis* FB-4. The results indicated that FB-4 significantly inhibited the mycelial growth of the pathogen. Treatment with FB-4 CFS or VOCs caused obvious hyphal malformation, suggesting that the strain secretes antifungal substances that disrupt the structural components of fungal cell walls. This aligns with previous reports on the antimicrobial potential of this species [36,37]. Specifically, the undiluted CFS of FB-4 inhibited conidial germination of the pathogen by 88.21%; treatment with 10% CFS resulted in smaller and denser mycelial pellets and a 93.2% reduction in mycelial dry weight; In detached twig assays, the undiluted CFS provided protective and curative efficacies of 74.49% and 58.97%, respectively. These results are consistent with the study by Ye et al., in which *B. velezensis* ZN-S10 affected conidial germination and mycelial growth of *Colletotrichum changpingense* and effectively reduced lesion area [38]. Similarly, Zhao et al. found that CFS from *B. velezensis* A4 inhibited spore germination, germ tube elongation, and mycelial growth of *Botrytis cinerea* in vitro and attenuated its pathogenicity on four tested fruits [39]. These consistent findings fully demonstrate the high efficacy of *B. velezensis* FB-4 in suppressing the canker pathogen. Although the detached twig experiments demonstrated the promising biocontrol potential of *Bacillus velezensis* FB-4 against Korla fragrant pear canker, field trials are still needed to validate its practical efficacy under real orchard conditions. Factors such as temperature, humidity, microbial interactions, and tree physiological status in the field may influence the colonization and antagonistic activity of the strain. Therefore, further multi-year and multi-site field trials in major pear-producing areas are warranted to systematically evaluate the disease control efficacy of FB-4 using different application regimes, as well as its impact on tree growth and fruit quality.

Bacterial metabolism produces a wide range of volatile organic compounds (VOCs), with individual strains typically emitting between 1 and 30 different compounds [40,41]. In this study, three putative antifungal VOCs were identified from *Bacillus velezensis* FB-4, which may account for the physiological abnormalities observed in *Cytospora pyri*. Specifically, acetaldehyde—a small aldehyde molecule—has been shown to significantly inhibit spore germination and mycelial growth of phytopathogenic fungi, exhibiting broad-spectrum biological activity [42,43]. Hexadecane, another VOC produced by strain FB-4, possesses antifungal and antioxidant properties, in addition to other reported activities such as insecticidal, nematicidal, and hypocholesterolemic effects [44,45]. Notably, 3-hydroxydodecanoic acid exerts potent inhibitory effects against various filamentous phytopathogenic fungi. Its proposed mechanism involves disruption of the fungal cell membrane structure, leading to increased membrane permeability, leakage of intracellular electrolytes and proteins, and ultimately disorganization of the fungal cytoplasm [46,47]. Future work will focus on quantitative analysis, activity screening of pure compounds, and dose–response evaluations to rigorously verify these specific structure-function relationships.

Microbial agents possessing both antagonistic and plant growth-promoting activities hold greater practical value in sustainable agriculture [33]. *B. velezensis* strains are widely recognized for this dual functionality, promoting plant growth through various mechanisms such as nitrogen fixation, phosphate solubilization, production of indole-3-acetic acid (IAA), siderophore-mediated iron acquisition, and enhancement of chlorophyll content [30,32,36]. In this study, strain FB-4 exhibited several key plant growth-promoting traits, including the ability to produce IAA and siderophores and to solubilize phosphate, which likely contribute to its growth-promoting effects. Although strain FB-4 produced several hydrolytic enzymes (e.g., protease and cellulase) and siderophores, which may potentially contribute to pathogen suppression by degrading cell wall components or competing for iron, the direct role of these traits in the observed antifungal activity remains unclear. Further studies, such as gene disruption or purified enzyme assays, are required to elucidate their specific contributions.

Seed germination and pot experiments further confirmed its efficacy: treatment with FB-4 bacterial suspension significantly increased the germination rate of tomato seeds and improved growth parameters, including plant height, fresh weight, and dry weight, at all tested concentrations. These growth-promoting effects may be attributed to bacterially derived phytohormones such as IAA, as well as other bioactive metabolites commonly produced by *Bacillus* species [48]. While the primary objective of this study was to obtain a biocontrol agent against pear canker, the additional plant growth-promoting properties of FB-4 (e.g., IAA production, phosphate solubilization) could provide supplementary benefits in orchard settings by enhancing host vigor and nutrient uptake, which may indirectly contribute to disease tolerance. However, these growth-promoting effects are not directly involved in the antifungal mechanism against *Cytospora pyri*. In summary, these results position *B. velezensis* FB-4 as a promising multifunctional biocontrol agent, capable of not only suppressing pathogen growth but also directly promoting plant development, thereby supporting its potential integration into environmentally friendly crop management systems.

## 5. Conclusions

In this study, an endophytic bacterial strain, FB-4, exhibiting significant antagonistic activity against the pear *Valsa* canker pathogen *Cytospora pyri*, was isolated from the bark of healthy Korla fragrant pear trees and identified as *Bacillus velezensis*. The cell-free supernatant of this strain strongly inhibited mycelial growth and conidial germination of the pathogen. In detached-twig assays, it demonstrated both protective and curative effects against the disease. Active volatile constituents emitted by FB-4 were identified, providing a basis for subsequent quantitative, field, and mechanistic studies. Furthermore, strain FB-4 also possessed plant growth-promoting traits. While the latter are not essential for its antifungal action, they provide added value for potential application in integrated disease management. These findings indicate that *B. velezensis* FB-4 is a promising multifunctional microbial inoculant that could be employed in integrated management of pear *Valsa* canker and contribute to the sustainable production of Korla fragrant pear.

## Figures and Tables

**Table 1:** Inhibition rates of 21 antagonistic endophytes against *Cytospora pyri*.

Endophytic Strains	Inhibition (%)	Scientific Name	Sequence Similarity (%)	Accession Number
FB-4	85.51 ± 0.09 a	*Bacillus velezensis*	99.86	PX690612
NF-1	85.24 ± 0.09 b	*Aspergillus flavus*	99.98	PP837541
FB-19	82.20 ± 0.08 c	*Pseudomonas chlororaphis*	99.02	PP837457
TB-1	80.93 ± 0.09 d	*Streptomyces rochei*	99.51	PP935386
TB-21	76.74 ± 0.10 e	*Bacillus velezensis*	100	PP837464
WB-8	76.55 ± 0.11 e	*Bacillus subtilis*	100	PP837450
KB-2	75.65 ± 2.67 f	*Bacillus amyloliquefaciens*	100	PP935372
KF-12	75.51 ± 0.14 f	*Chaetomium elatum*	99.45	PP837506
WB-16	74.69 ± 0.11 g	*Paenibacillus farraposensis*	98.89	PP935380
FB-32	73.66 ± 0.07 h	*Bacillus tequilensis*	99.17	PP935364
KB-7	72.20 ± 0.07 i	*Pseudomonas uvaldensis*	99.25	PP837466
NB-11	71.22 ± 0.07 j	*Bacillus albus*	99.93	PP785796
NB-9	70.85 ± 0.13 k	*Streptomyces albidoflavus*	99.54	PP837450
TB-36	69.18 ± 0.13 L	*Bacillus velezensis*	99.13	PP837460
KB-29	69.12 ± 0.07 lm	*Bacillus pumilus*	100	PP785799
WB-3	68.93 ± 0.21 m	*Stenotrophomonas rhizophila*	100	PP837451
NB-25	66.09 ± 0.19 n	*Streptomyces flavofungini*	99.16	PP935379
FB-7	65.91 ± 0.15 n	*Bacillus amyloliquefaciens*	100	PP837463
NB-31	64.45 ± 0.30 o	*Bacillus atrophaeus*	99.31	PP935373
TB-20	63.76 ± 0.34 p	*Metabacillus idriensis*	100	PP935381
KB-9	63.31 ± 0.17 q	*Streptomyces murinus*	99.93	PP837461

Data are the mean ± SD. Different lowercase letters indicate statistically significant differences (*p* < 0.05) according to Tukey’s HSD test.

**Table 2:** Biochemical and physiological characteristics of strain FB-4.

Test Items	Test Result
Voges-Proskauer	+
Nitrate reductase	+
Citrate	+
Gelatin liquefaction	+
Urease	−
Sucrose	+
Xylose	+
Maltose	+
Mannitol	+

Note: “+” indicates a positive result; “−“ indicates a negative result.

**Table 3:** Lesion length and control efficacy on detached twigs of Korla fragrant pear under different treatments.

Treatment	Lesion Length/mm	Control Effect/%
Inoculation with *Cytospora pyri* only	62.71 ± 4.68 a	—
Inoculation with *C. pyri* followed by application of CFS from *Bacillus velezensis* FB-4	25.57 ± 2.92 b	58.97 ± 6.43 b
Application of CFS from antagonistic *B. velezensis* FB-4 followed by inoculation with *C. pyri*	15.78 ± 3.15 c	74.49 ± 6.49 a
Mock-inoculated control (PDA medium only)	0.00 ± 0.00 d	—

Data are presented as the mean ± standard deviation. Different lowercase letters indicate statistically significant differences (*p* < 0.05) according to Tukey’s HSD test.

**Table 4:** Volatile organic compounds identified from *Bacillus velezensis* FB-4 with potential antifungal activity.

No.	Compound	Rel. Area %	CAS Number	Retention Time/min
1	Acetaldehyde	0.10	75-07-0	6.521
2	Hexadecane	0.06	544-76-3	15.355
3	3-hydroxydodecanoic acid	0.03	1883-13-2	38.756

**Table 5:** Effect of *Bacillus velezensis* FB-4 on tomato growth promotion.

Treatment	Plant Height (cm)	Fresh Weight (g)	Dry Weight (g)
CK	13.95 ± 2.58 c	3.68 ± 0.63 c	0.16 ± 0.03 d
1 × FB-4	16.89 ± 3.34 b	7.08 ± 0.78 b	0.39 ± 0.05 c
10 × FB-4	19.02 ± 3.25 ab	7.73 ± 0.8 b	0.48 ± 0.08 b
50 × FB-4	20.83 ± 3.41 a	9.15 ± 1.93 a	0.56 ± 0.13 a

Growth performance of tomato seedlings after soil drenching with different concentrations of FB-4 cell suspension [1 × (1 × 10^8^ CFU/mL), 10× (1 × 10^9^ CFU/mL), 50× (5 × 10^9^ CFU/mL)] or sterile water (control) for 20 days. Data are presented as mean ± SD (n = 12 biological replicates). Different lowercase letters in the same column indicate statistically significant differences (*p* < 0.05) according to Tukey’s HSD test.

## Data Availability

The original contributions presented in this study are included in the article/Supplementary Material. Further inquiries can be directed to the corresponding authors.

## Supplementary Materials

The following supporting information can be downloaded at: https://www.mdpi.com/article/10.3390/jof12050349/s1, Supplementary Material S1: *Bacillus velezensis* FB-4 VOCs; Supplementary Material S2: LB VOCs.
